# Malignant glomus tumor of jejunum with liver and peritoneum metastasis: a rare case report

**DOI:** 10.3389/fonc.2024.1519968

**Published:** 2025-01-17

**Authors:** Shilan Peng, Nan Yang, Zifan Xu, Guomiao Su, Xiaoyu Tuo, Shiyue Liu, Zi Lei, Guoqing Pan

**Affiliations:** ^1^ Department of Pathology, First Affiliated Hospital of Kunming Medical University, Kunming, China; ^2^ College of The First Clinical Medicine, Gansu University of Chinese Medicine, Lanzhou, China

**Keywords:** jejunum, malignant, glomus tumor, case report, diagnose

## Abstract

Glomus tumors (GTs) are rare mesenchymal neoplasms that occur predominantly on the subungual region of the distal extremities and are rarely seen in visceral organs such as the gastrointestinal tract. Malignant glomus tumors (MGTs) is even more rare, comprising less than 1% of all GTs. We reported an 82-year-old female patient with MGTs of the jejunum, accompanied by metastases to the liver and peritoneum. The patient presented with a primary complaint of epigastric pain with nausea and acid reflux for two months. Computed tomography scan revealed a prominently enhanced, inhomogeneous-density mass in the jejunum, the nature of which—benign or malignant—could not initially be determined. Postoperative pathological diagnosis confirmed the lesion to be a jejunal MGT. Regrettably, the patient declined additional treatment, subsequently developing liver and peritoneal metastases one year later. She eventually died within 18 months of initial diagnosis. This report summarizes the clinical and histopathological features of jejunal MGTs with the aim of increasing awareness among clinicians and pathologists regarding this disease.

## Introduction

Glomus tumors (GTs) are rare, generally benign neoplasms of mesenchymal origin, most likely arising from mutated smooth muscle cells of neurovascular glomus body (1). GTs constitute approximately 2% of all soft tissue tumors and most commonly occur in adults aged 20 to 40, with an equal frequency in both the sexes (2). Although GTs can develop in any part of the body, they are most frequently found in the subcutis of extremities, particularly the subnail region. The gastrointestinal tract is not the predilection site of GTs, especially in the small intestine where they are rare (3). GTs lack the specificity in imaging examination, and diagnosis of GTs is primarily based on histopathological examination following an operation (4). The primary treatment modality for GTs involves surgical excision with clear margins to mitigate the risk of recurrence, and complete excision is typically curative for benign GTs. Malignant glomus tumours (MGTs), which account for less than 1% of all GTs (5), are highly invasive and require more aggressive treatment, including extensive surgical resection, and may hardly benefit from low-dose adjuvant radiotherapy or chemotherapy (3). The case reported in this article presents an in-depth analysis of a patient suffered from MGTs with metastasis found in liver and peritoneum, specifically highlighting the rarity of its occurrence in the jejunum area. To the best of our knowledge, this is the second documented instance of MGT manifesting in this anatomical location.

## Case presentation

**Figure 1:**
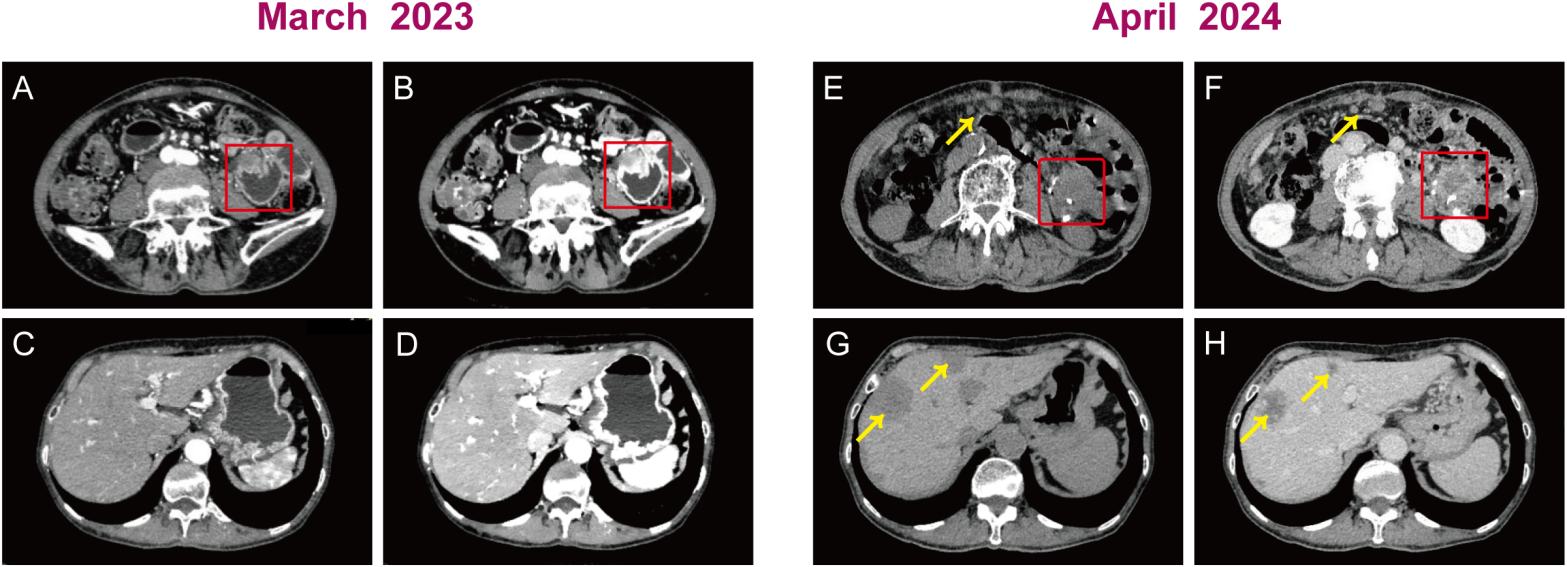
Plain and contrast enhanced computed tomography scans of the abdomen. Preoperative images: **(A, B)** A uneven thickening of the jejunum in the left epigastric region (red box). **(C, D)** The liver had no obvious abnormalities. Postoperative images: **(E, F)** A jejunal anastomotic mass with 3.1 cm x 2.4cm of diameter (red box) and multiple nodular masses in the abdominopelvic region, omentum (yellow arrow). **(G, H)** Scattered circular mass in the liver (yellow arrow).

In March 2023, an 82-year-old Chinese woman with a history of hypertension came in with a chief complaint of epigastric pain with nausea and acid reflux for two months. A vomiting episode with gastric contents had occurred a week earlier. Despite treatment with omeprazole, abdominal pain persisted. Physical examination revealed mild and diffuse tenderness in the abdomen, particularly 1~2cm below the xiphoid. Computed tomography scan of the abdomen showed localized uneven thickening of the jejunum in the left epigastric region, suspicious for a neoplastic lesion (**Figures 1A, B**). The mass in the jejunum caused dilatation and effusion in the stomach and duodenum on the images. There are no obvious abnormalities in the liver except for hepatic cysts (**Figures 1C, D**). The differential diagnosis was very difficult, given the non-specific clinical features and radiologic information.

**Figure 2:**
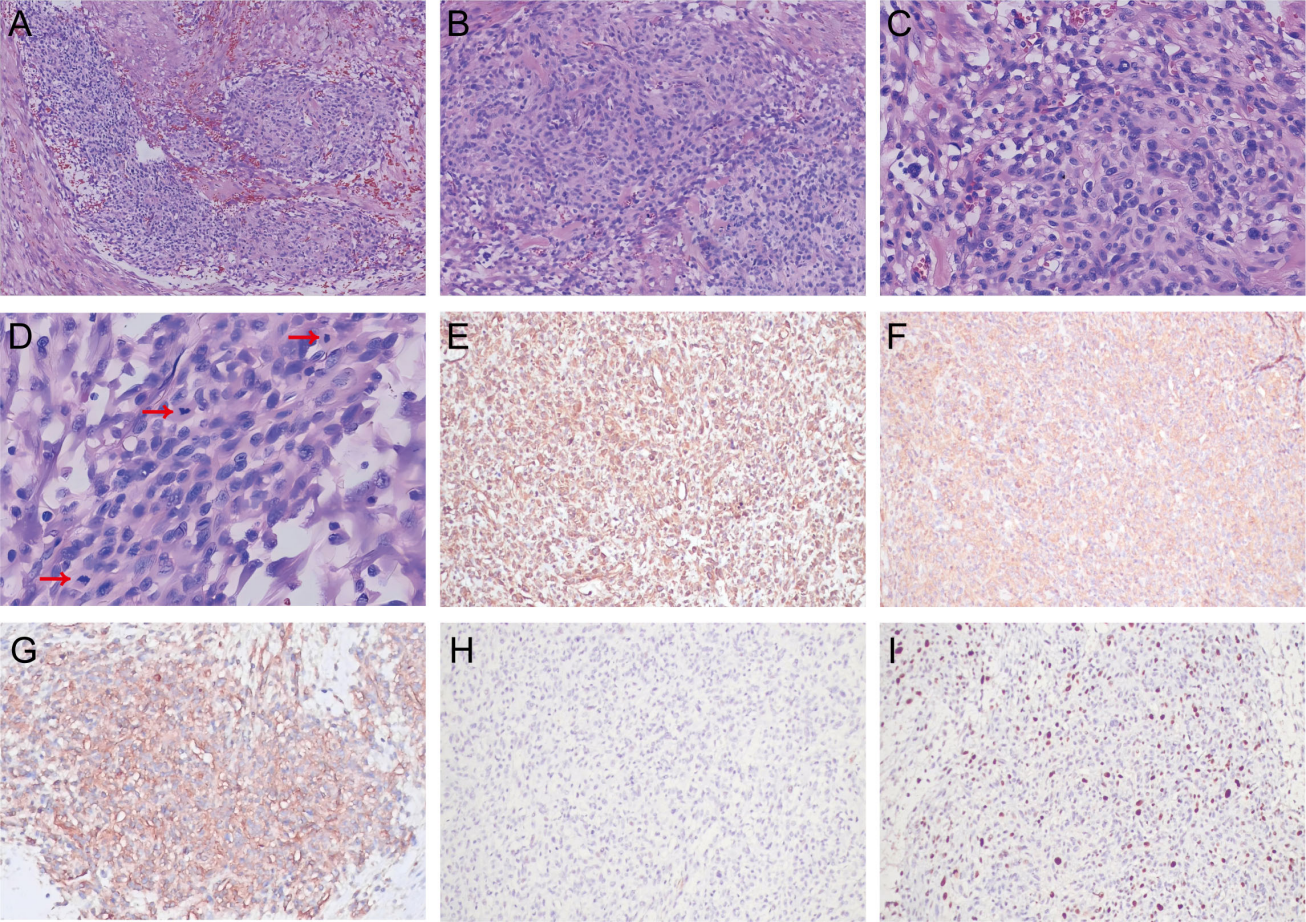
Histological analysis of malignant glomus tumor in the jejunum. Hematoxylin–eosin (HE): **(A)** The tumor cells showed a nested growth pattern (magnification, 40×). **(B)** Relatively uniform tumor cells distributed around mildly dilated, thin-walled vessels (magnification, 100×). **(C)** The tumor cells exhibited an epithelioid morphology, round to oval nuclei and obvious nuclear atypia (magnification, 200×). **(D)** There was increased mitosis (red arrow) (magnification, 400×). Immunohistochemistry: **(E)** Vimetin positivity. **(F)** Strong positive for smooth muscle actin. **(G)** Collagen IV positivity. **(H)** Negative for desmin. **(I)** Ki-67 proliferative index of 20%.

Following imaging examination, the patient was promptly administered acid suppression therapy and fluid replacement to alleviate symptoms of epigastric pain accompanied by acid reflux. Despite five days of symptomatic treatment, there was no improvement in the patient’s symptoms, and the frequency of vomiting due to intestinal obstruction had increased. On the sixth day of hospitalization, surgical excision was performed in response to the obstructive condition and the necessity for urgent intervention. A 9 cm long and 3 cm diameter intestinal canal was removed and a 4.5 cm x 2.0 cm x 1.8 cm nodular formation was observed in the submucosa, with evidence of invasion into the entire intestinal wall and breach of the plasma membrane layer. Moreover, intraoperative observations revealed tumor invasion into the mesentery of the descending colon. Histopathological examination showed a nested growth pattern of relatively uniform tumor cells, interspersed with occasionally mildly dilated, thin-walled vessels encircled by tumor cells (**Figures 2A, B**). The cells exhibited an epithelioid morphology, characterized by eosinophilic cytoplasm and round to oval nuclei with inconspicuous nucleoli. There was obvious nuclear atypia and mitosis like 15/50 HPF (**Figures 2C, D**). Immunohistochemical analysis revealed strong positivity for vimetin (**Figure 2E**), smooth muscle actin (**Figure 2F**) and collagen IV (**Figure 2G**), with a Ki-67 proliferative index of 20% (**Figure 2I**). It was negative for desmin (**Figure 2H**). Multiple other antibodies were stained and the results showed negativity for pancytokeratin, chromogranin A, synaptophysin, calretinin, CD56, CD34, CD117, DOG-1, S-100, LCA, WT1. Based on the clinical, radiologic, and pathologic information, the patient was diagnosed with malignant glomus tumor.

Post-operatively, there was a marked improvement in the patient’s abdominal discomfort and episodes of emesis. In light of the patient’s advanced age, physical condition, and her own wishes, no additional systemic therapy was administered following the surgical procedure. Treatment comprised routine therapy of gastrointestinal tract protection and anti-symptomatic measures. Given the aggressive biology of the tumor, the patient was monitored every month postoperatively. In April 2024, postoperative CT revealed jejunal anastomotic mass (**Figures 1E, F**) with multiple metastases found in the abdominopelvic region, omentum, and liver (**Figures 1G, H**), suggestive of recurrent neoplastic disease. The patient offered to discontinue treatment and subsequently developed more generalized weakness and profound cachexia. She eventually died within 18 months of initial diagnosis.

## Discussion

Glomus bodies are normally situated within the stratum reticularis of the dermis and are predominantly found in the subungual region, lateral aspects of the digits, and the palmar surface (6). Their presence in visceral locations, such as the stomach, intestines, or lungs, is exceedingly rare due to the scarcity or absence of glomus bodies in these organs (7). Although a few cases of GT have been occasionally found in the gastrointestinal tract, they are mostly located in the stomach rather than the intestine (8–10). Intestinal GTs occurred more frequently in the ileum and colon (3, 8). To date, only two GT originating from the jejunum have been described, comprising one benign GT and one MGT (11, 12). In this context, we present the second reported case of primary jejunal MGT with metastasis to the liver and peritoneum.

Intestinal MGTs exhibit a wide spectrum of clinical manifestations, ranging from asymptomatic presentations to symptoms such as nausea, vomiting, and anemia, progressing to more severe gastrointestinal manifestations (13–15). Endoscopically, intestinal MGTs are characterized by submucosal lesions that may present with either normal mucosal surfaces or ulceration (16). Computed tomography scan generally shows a gastrointestinal submucosal tumor with strong enhancement in the arterial phase and prolonged enhancement in the delayed phase, indicating its hypervascular characteristics (14, 17, 18). This feature is consistent with our case. In our instance, computed tomography scan revealed a prominently enhanced, inhomogeneous-density mass in the jejunum. Nevertheless, these radiologic findings were not enough to differentiate MGTs from other subepithelial lesions. Coupled with the nonspecific nature of the clinical symptoms and endoscopic features, these lack of specificity complicates the accurate diagnosis.

Histologically, although intestinal GTs share similarities with other parts of the body, the rarity of intestinal GTs contributes to the frequent misdiagnosis of GTs in such locations. GTs consist of modified perivascular smooth muscle cells organized into sheets and nests, which are densely populated with blood vessels of varying sizes. The cells exhibit a round and epithelioid morphology (19). Occasional isolated nests of glomus cells can be identified outside its boundaries and proliferate around vessels at the periphery of the main tumor. A hemangiopericytomatous vascular arrangement may be observed once in a while. Despite their epithelioid morphology and close association with vessels, glomus cells do not express epithelial or endothelial markers. The cells show prominent staining with smooth muscle markers, such as smooth muscle actin, h-caldesmon (7, 20, 21). Furthermore, most GTs were positive for collagen IV, calponin, and vimentin, and were negative for pancytokeratin, chromogranin A, synaptophysin, CD117 and S-100. Immunoreactivity for desmin is variable, with patterns ranging from absent to focal positivity (22). In our case, the presence of vimentin-positive staining confirms the mesenchymal nature of the tumor. Concurrently, the strong positivity for smooth muscle actin, alongside the negativity for CD117, chromogranin A, and synaptophysin, suggests that the tumor is derived from smooth muscle cells rather than from interstitial cells of Cajal or neuroendocrine cells. Furthermore, desmin negativity coupled with collagen IV positivity is highly suggestive of GTs. Studies have shown that rearrangements of NOTCH genes occur in more than half of GTs, with NOTCH2-MIR143 being the most common fusion (23). Moreover, all 5 examples of MGTs included in a study by Mosquera et al. showed the presence of NOTCH-gene rearrangements (24). Regretfully, the patient in this case was not tested for NOTCH-gene rearrangement. Although occasional cases of the BRAF V600E mutation have been identified in MGTs and uncertein malignant potential GTs (25), there are no specific cytogenetic findings that support the diagnosis of GTs.

The main challenge in diagnosing intestinal GTs is classifying the tumour as benign, malignant or of uncertain malignant potential. The diagnosis of MGTs is usually made according to Folpe’s study (26), which summarised the pathological features of 52 cases. The diagnostic criteria proposed by the authors included the presence of tumors in deep anatomical locations, a size exceeding 2 cm, atypical mitotic figures, moderate to high nuclear grades, and a mitotic activity of ≥ 5/50 HPFs (400×). Later results showed that tumors with a size exceeding 2 cm and a deep anatomical location have also been observed in GTs of uncertain malignant potential. Consequently, the WHO (2016) outlines that the diagnostic criteria for MGTs should fulfill at least one of the following conditions (1): the presence of pronounced nuclear atypia in tumor cells, accompanied by varying degrees of mitotic activity; or (2) the observation of atypical mitotic figures. According to these criteria, this case with serosal invasion, large tumor sizes (maximum diameters of 4.5cm), high nuclear grades and increased mitotic activity (15/50 HPFs) met the diagnostic criteria for MGTs. These characteristics are particularly associated with the risk of metastasis (7), which is confirmed by the subsequent progression of the patient’s disease. Typically, MGTs are classified into two distinct categories: those that exhibit morphological similarities to leiomyosarcomas or fibrosarcomas, and those that structurally resemble benign glomeruli but contain conspicuous nests of highly malignant round cell components. The case discussed in this article pertains to the latter category. However, it is not currently known whether there is a difference in the degree of malignancy between these two subtypes.

MGTs of the jejunum are extremely rare, and it is therefore necessary to rule out other diseases in patients presenting with a pelvic-abdominal mass. From a pathological standpoint, the major differential diagnoses for intestinal MGTs includes gastrointestinal stromal tumors (GISTs), neuro-endocrine tumors (NETs) and leiomyomas/leiomyosarcomas. GISTs and NETs may show some similar morphological manifestations to this case of MGT. The two types of tumors are usually easily distinguishable by immunohistochemistry. GISTs stained positively for CD117 and CD34 (27). NETs demonstrated significant positive staining for the neuroendocrine markers chromogranin A, neuron-specific enolase, synaptophysin, and CD56 (28). In contrast, GTs generally showed negative staining for CD117 and neuroendocrine markers, with only a minority of cases displaying positivity for CD34. While focal areas resembling leiomyomas/leiomyosarcomas may be observed in some MGTs, the former characteristically consist of interlacing fascicles of spindle cells, with nuclei that are elongated and possess blunt ends, often described as “cigar-shaped”. The vascularization within leiomyomas is less pronounced, and the number of mitotic figures is lower than in MGTs. In contrast to MGTs, leiomyosarcoma cells exhibit larger volumes, more abundant eosinophilic cytoplasm, thicker vessel walls, and may occasionally display tumor necrosis (26). Furthermore, leiomyomas and leiomyosarcomas typically exhibit diffuse and strong positive expression of smooth muscle actin and desmin (29), aiding in differential diagnosis.

MGTs is highly aggressive, characterized by high rates of recurrence and metastasis. Owing to the exceptionally low incidence of intestinal GTs and the lack of comprehensive clinical data, it is difficult to identify an effective treatment for MGTs of the intestine (3). Current therapeutic strategies predominantly encompass wide local excision, with the primary objective of attaining negative margins, a critical factor in minimizing the risk of recurrence and metastasis. In instances where the tumor is situated in anatomically critical regions or is extensive, more complex surgical approaches may be necessary. Nonetheless, the surgery should aim to prioritize patient survival over the preservation of functional outcomes (30). There is limited data on the effectiveness of adjuvant chemotherapy for MGTs. Chemotherapy is generally administered in instances of metastatic disease, and treatment strategies often resemble those used in the management of soft tissue sarcoma (31). A subset of patients undergoes postoperative adjuvant chemotherapy, although the response to such treatment is generally suboptimal (32). In general, due to the mobile nature of the target and other radiobiological concerns, adjuvant radiotherapy is typically not considered for resected jejunal or small bowel tumors. However, palliative radiotherapy may be exceptionally and cautiously considered for intact masses if they are symptomatic. However, the long-term advantages of radiotherapy for MGTs are not well established, as some studies indicate limited effectiveness (33). In this particular case, the patient was offered the radical jejunectomy. However, the patient declined additional treatment and subsequently developed a postoperative recurrence and metastases to the liver and peritoneum one year later. Consequently, this case does not contribute effectively to the development of therapeutic guidelines for rare intestinal MGTs. Note that the recurrence pattern observed in this case does not significantly differ from that of other common malignant tumors of the jejunum, such as jejunal adenocarcinoma. It is characterized by recurrence in proximity to the surgical resection site and distant metastases to the lungs, liver, peritoneum, and other regions.

Metastasis is a critical determinant of poor prognosis in MGTs. Prior research indicates that 62.5% (10 out of 16) of MGTs originating from the trachea, bronchus, or lung were associated with distant metastases, with six patients died during a 60-month follow-up period (3). Consistent with this finding, the patient in our case died four months after the development of hepatic and peritoneal metastases. One study showed that the presence of tumoural necrosis negatively correlated with disease outcome (34). In addition, factors influencing the prognosis of MGTs include tumor size, mitotic count, and vascular and nerve invasion (9). Consequently, it is essential that pathologists incorporate these factors in a standardized manner within diagnostic reports. Simultaneously, clinicians must rigorously evaluate these variables throughout the treatment and monitoring processes.

## Conclusion

In addition to their infrequent morbidity, MGTs exhibit a lack of specificity in clinical manifestations, imaging, and endoscopic features, which can lead to missed or misdiagnosed cases. We present a case of jejunal MGT with a high degree of malignancy and multiorgan metastases. This case underscores the importance of considering MGTs as a differential diagnosis when encountering irregular masses in the jejunum. Accurate diagnosis necessitates meticulous histological examination and immunostaining for relevant markers. MGTs are highly aggressive and associated with a poor prognosis. Complete surgical resection represents an effective radical intervention for intestinal MGTs. Nevertheless, the paucity of relevant case reports has resulted in an absence of well-defined and efficacious postoperative treatment protocols. Consequently, extended clinical follow-up is essential to gather additional evidence and enhance the assessment of the efficacy of postoperative treatment strategies.

## Data Availability

The original contributions presented in the study are included in the article/supplementary material. Further inquiries can be directed to the corresponding authors.
